# Combined Insults of a MASH Diet and Alcohol Binges Activate Intercellular Communication and Neutrophil Recruitment via the NLRP3-IL-1β Axis in the Liver

**DOI:** 10.3390/cells13110960

**Published:** 2024-06-01

**Authors:** Mrigya Babuta, Prashanth Thevkar Nagesh, Aditi Ashish Datta, Victoria Remotti, Yuan Zhuang, Jeeval Mehta, Francesca Lami, Yanbo Wang, Gyongyi Szabo

**Affiliations:** Department of Medicine, Division of Gastroenterology, Beth Israel Deaconess Medical Center and Harvard Medical School, Boston, MA 02215, USA; mbabuta@bidmc.harvard.edu (M.B.);

**Keywords:** NETs, MetALD, monocyte/macrophage, steatosis, inflammation, MASH

## Abstract

Binge drinking in obese patients positively correlates with accelerated liver damage and liver-related death. However, the underlying mechanism and the effect of alcohol use on the progression of metabolic-dysfunction-associated steatotic liver disease (MASLD) remain unexplored. Here, we show that short-term feeding of a metabolic-dysfunction-associated steatohepatitis (MASH) diet plus daily acute alcohol binges for three days induce liver injury and activation of the NLRP3 inflammasome. We identify that a MASH diet plus acute alcohol binges promote liver inflammation via increased infiltration of monocyte-derived macrophages, neutrophil recruitment, and NET release in the liver. Our results suggest that both monocyte-derived macrophages and neutrophils are activated via NLRP3, while the administration of MCC950, an NLRP3 inhibitor, dampens these effects.In this study, we reveal important intercellular communication between hepatocytes and neutrophils. We discover that the MASH diet plus alcohol induces IL-1β via NLRP3 activation and that IL-1β acts on hepatocytes and promotes the production of CXCL1 and LCN2. In turn, the increase in these neutrophils recruits chemokines and causes further infiltration and activation of neutrophils in the liver. In vivo administration of the NLRP3 inhibitor, MCC950, improves the early phase of MetALD by preventing liver damage, steatosis, inflammation, and immune cells recruitment.

## 1. Introduction

Liver diseases are a significant public health concern, causing a staggering two million deaths every year across the globe [1]. Alcohol-associated liver disease (ALD) and metabolic-dysfunction-associated steatotic liver disease/metabolic-dysfunction-associated steatohepatitis (MASLD/MASH) are the leading causes of chronic liver disease [1,2,3]. Alcohol use disorder is prevalent in approximately 5.1% (283 million people) of the global population and contributes to approximately 50% of liver-related deaths [1,2,3].

Heavy alcohol consumption combined with obesity has increased by 72% from 1999–2000 until 2017–2020 and its poses a considerable health burden in the United States [4]. Several studies have indicated exacerbation of ALD in obese patients [5,6,7,8,9]. In one such study performed in France, ALD patients with metabolic syndrome showed higher overall mortality [8]. Similarly, the UK Biobank study showed that patients with a high waist circumference who reported alcohol consumption had a greater liver disease incidence and mortality [10]. Thus, alcohol use in metabolic syndrome can serve as a prognostic cofactor for long-term morbidity and mortality [7,8,11]. Therefore, recently, a novel category known as MetALD was introduced to identify individuals who exhibit the characteristics of MASLD and have an alcohol consumption of greater than 20–50 g/day for females and 20–60 g/day for males [12].

The pathogenesis of most chronic liver diseases involves the activation of a proinflammatory cascade in the liver [9,13,14]. Inflammation is induced by both pathogen-associated molecular pattern molecules (PAMPs) and damage-associated molecular patterns (DAMPs). PAMPs are derived from intestinal dysbiosis and/or gut leakiness, while DAMPs are derived from dying/damaged hepatocytes and other cells [13,14,15]. DAMPs and PAMPs are sensed by pattern recognition receptors such as Toll-like receptors (TLRs) or intracellular inflammasome sensors expressed in innate immune cells such as monocytes and macrophages, which initiate the inflammatory responses [13,14,15].

Inflammasomes are multiprotein cytoplasmic complexes that are innate immune sensors implicated in several chronic inflammatory diseases [13,16,17,18]. Nucleotide-binding domain, leucine-rich-containing family, pyrin-domain-containing-3 (NLRP3) is one of the most studied inflammasomes and is highly expressed in Kupffer cells and other immune cells in the liver but is less expressed on hepatocytes and stellate cells [13]. The NLRP3 inflammasome comprises an inflammasome sensor NLRP3, an apoptosis-associated speck-like protein containing a caspase recruitment domain (ASC), which acts as an adapter, and the precursor pro-caspase-1 [13,16,17,18]. Upon stimulation, NLRP3 oligomerizes and, via its pyrin domain, recruits ASC to form the inflammasome complex, which cleaves caspase-1 and, in turn, activates the proinflammatory cytokines interleukin-1β (IL-1β) and interleukin-18 (IL-18) [13]. Activation of NLRP3 can also lead to pyroptotic cell death when caspase-11 cleaves gasdermin-D, forming pores in the cell membrane [13]. NLRP3 activation is associated with the progression of ALD as ASC speck formation upon NLRP3 activation propagates the systemic and liver inflammation even after the cessation of alcohol use [13,19].

Increased inflammation and damaged hepatocytes recruit immune cells in the liver, especially macrophages and neutrophils. Neutrophil infiltration is considered as a hallmark of ALD pathogenesis [20,21]. It has recently been shown that increased neutrophil infiltration in ALD results in spontaneous neutrophil extracellular trap (NET) formation. The alcohol-induced decreased capacity of macrophages to clear these NETs leads to persistent liver inflammation and injury by NETs [22,23,24]. A recent study also showed that alcohol-induced NETs activate hepatic stellate cells (HSCs) via NLRP3 and accelerate fibrosis in a murine model of MetALD [25].

We previously showed that the high fat–cholesterol–sucrose diet results in progression from steatosis to steatohepatitis, culminating in HCC, over the course of 12 to 48 weeks. Previous studies have shown that even short-term feeding of a high-fat diet (HFD) for three days results in insulin sensitivity and impairs glucose tolerance in mice [26,27,28,29]. Furthermore, combining three days of a high-fat diet with a single alcohol binge exacerbates neutrophil infiltration and liver damage as compared to a high-fat diet alone [23]. Interestingly, the results of long-term feeding of a HFD showed that it was only male mice that displayed robust steatohepatitis and inflammasome activation as compared to female mice, which had steatosis without inflammation [30,31].

In the current study, we describe that a short-term three-day murine model of a high fat–cholesterol–sucrose diet (hereafter referred to as a MASH diet) plus daily acute alcohol binges induce liver injury, NLRP3 activation, and neutrophil infiltration. We further report the role of the NLRP3-IL-1β axis in recruiting neutrophils and NET formation and demonstrate NLRP3 inhibition as a therapeutic target in the short-term feeding of a MASH diet plus acute alcohol binges.

## 2. Material and Method

### 2.1. Animal Care and Treatments

Nine- to ten-week-old male C57BL/6J mice were purchased from the Jackson Laboratory and housed in Beth Israel Deaconess Medical Center (BIDMC) in compliance with institutional guidelines. The mice were kept under a controlled atmosphere in 12 h light and dark cycles. All the additional procedures were approved by the BIDMC institutional animal care and use committee (IACUC, protocol #019-2019, #30-2022).

The mice were randomly divided into two groups and were either fed on standard laboratory chow diet or on a MASH diet (from Research Diets, Inc., New Brunswick, NJ, USA-D09061704) ad libitum for three days. The MASH diet was composed of high fat (33 gm%), high cholesterol (10 gm%), and high sucrose (208.4 gm%). The chow-fed mice received water gavage every day for three days and the MASH diet-fed mice received 5 g/kg alcohol gavage for three days. The mice were sacrificed 9 h after the final binge, and blood and liver samples were collected, processed immediately, and stored at −80 °C for further analyses.

For the MCC950 intervention, 9- to 10-week-old male C57BL/6J mice were either fed on a chow diet or a MASH diet for 3 days. The MASH diet-fed (ad libitum for three days) mice, which received 5 g/kg alcohol gavage every day for 3 days, were randomly divided into two group in which one group (n = 6–8) received vehicle and the other group (n = 6–8) received MCC950 via i.p. (10 mg/kg of body weight) (Cayman Chemicals) (n = 6–8). Vehicle or MCC950 i.p. injection was given 1–2 h before each alcohol gavage for three days. The mice were sacrificed 9 h after the final binge, and blood and liver samples were collected, processed immediately, and stored at −80 °C for further analyses. The animal experiments were reported using the ARRIVE reporting guidelines [32].

### 2.2. Neutrophil Isolation

Neutrophils from the mice were isolated from the bone marrow as described previously [33]. Briefly, bone marrow cells were collected from 8- to 10-week-old WT or NLRP3-KO mice by flushing cells from both the femur and tibia by using a 25-gauge needle and 12 cc syringe cells. Following this, the cells were centrifuged, and the MojoSort™ mouse neutrophil isolation kit was used, as per the manufacturer’s instruction, to isolate neutrophils via negative selection.

### 2.3. Neutrophil Extracellular Trap (NET) Formation Assay

Bone-marrow-derived mice neutrophils were isolated as described above. A total of 1 million neutrophils were used per experimental condition. WT or NLRP3-KO bone marrow neutrophils were treated with 50 mM ethanol or 330 μM palmitic acid (PA) for 4 h to test the alcohol- and PA-induced NET formation in the neutrophils. After removing the supernatant, the NETs at the bottom of the plate were treated with S7 DNase I for 15 min at 37 °C. The cell-free suspension was used to quantify the NET-associated neutrophil elastase according to the manufacturer’s instructions to quantify the extent of the NET formation.

### 2.4. Western Blot and ELISA

Western blotting was performed on the total proteins extracted from the mouse livers. Equal amounts of total protein were resolved on a 10–15% acrylamide gel and transferred to nitrocellulose membranes. The membranes were blocked in 5% BSA and then probed with specific antibodies for caspase-1 (Abcam, Waltham, MA, USA-ab179515), gasdermin D (Abcam, Waltham, MA, USA-ab219800), and IL-1β (GeneTex, Irvine, CA, USA-GTX74034), as indicated.

This study used the Legend Max™ Mouse CXCL1 ELISA Kit (BioLegend, San Diego, CA, USA #447507), Mouse Lipocalin-2/NGAL Quantikine ELISA Kit (R & D Systems, Minneapolis, MN, USA #MLCN20) for serum, supernatant and liver lysates; Mouse Neutrophil Elastase/ELA2 Quantikine ELISA Kit (R & D Systems #MELA20) for serum and liver lysates; Citrullinated Histone H3 (Clone 11D3) ELISA Kit (Cayman Chemical Company, Ann Arbor, MI, USA #501620) for liver lysates; Mouse IL-1 beta/IL-1F2 Quantikine ELISA Kit (R & D Systems #MLB00C) for liver lysates; and Mouse IL-1 beta/IL-1F2 Quantikine HS ELISA Kit (R & D Systems, MN, USA#MHSLB00) for serum. All the ELISAs were performed as per the manufacturer’s protocol. A total of 30 μg of total liver lysates and mice serum diluted to 1:5–1:10 in the appropriate buffer was used for the ELISA.

### 2.5. Flow Cytometry

Liver immune cells were isolated as described previously [34]. Briefly, immune cells were incubated with TruStain FcX™ (BioLegend, San Diego, CA, USA 101320, anti-mouse CD16/32) antibody for 5 min on ice followed by 1X with FACS buffer (HSBSS + 2%FBS). The cells were then incubated with a cocktail of antibodies (1:100 dilution) for 30 min on ice: APC/Cyanine7 anti-mouse CD45 antibody (BioLegend, San Diego, CA, USA 157204, clone S18009F), Brilliant Violet 421™ anti-mouse F4/80 antibody (BioLegend, San Diego, CA, USA 123132, clone BM8), Brilliant Violet 785™ anti-mouse Ly-6G antibody (BioLegend, San Diego, CA, USA 127645, clone 1A8), Brilliant Violet 605™ anti-mouse CD86 antibody (BioLegend, San Diego, CA, USA 105037, clone GL-1), FITC anti-mouse CD206 (MMR) antibody (BioLegend, San Diego, CA, USA 141704, clone C068C2), Brilliant Violet 711™ anti-mouse/human CD11b antibody (BioLegend, San Diego, CA, USA 101242, clone M1/70), and a Zombie NIR™ Fixable Viability Kit (BioLegend, San Diego, CA, USA Cat#423106). After 30 min, all the samples were washed once with FACS buffer and incubated with 4% PFA for 10 min at room temperature in the dark. Following fixing, the cells were washed twice and resuspended in FACS buffer. The samples were run in an Aurora Spectral Flow Cytometer (Cytek) and the data analysis was performed using Flowjo version 8.8.7 software.

### 2.6. RNA Extraction and qPCR

RNA was extracted using the RNeasy Kit (Qiagen, Germantown, MD, USA) with on-column DNase digestion. The concentration was determined using a Nanodrop 2000 (Thermo Scientific, Waltham, MA, USA), and 1 μg RNA was used for the cDNA reverse transcription (BioRad, Hercules, CA, USA). The quantitative real-time polymerase chain reaction (qPCR) was completed using SYBR Green polymerase (BioRad, CA, USA), and the expression was measured on a BioRad CFX96 Real-Time System. The qPCR primers are listed in, and the expression was quantified using the 2^−ΔΔCt^ method (Table 1).

## 3. Results

### 3.1. Short-Term Feeding of MASH Diet and Alcohol Binges Promote Liver Injury and Steatosis

**Figure 1:**
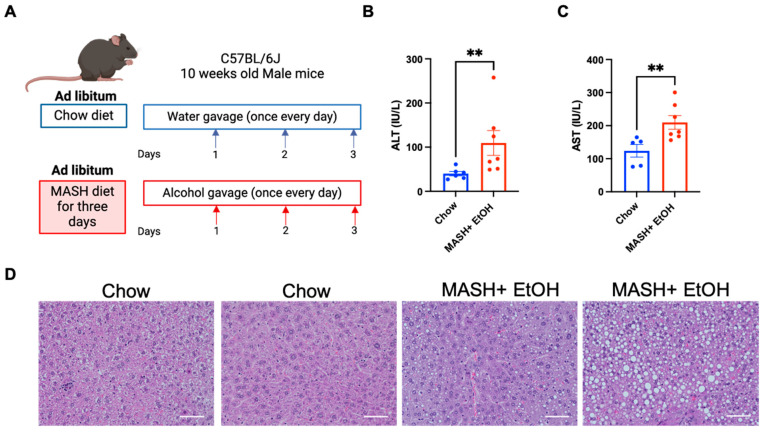
Short-term feeding of a MASH diet and acute alcohol binges promote liver injury and steatosis. (**A**) Feeding schematics for combined liver injury. C57BL/6 wild-type (WT) mice (n = 6–8) were fed on a MASH diet plus alcohol binges with a standard laboratory chow diet as a control for three days. (**B**,**C**) ALT and AST levels were measured from serum. (**D**) Formalin-fixed liver sections were stained with hematoxylin and eosin and representative slides are shown, scale bar = 50 μm. ** *p* < 0.005.

To understand the effects of acute alcohol binges combined with a high fat–cholesterol–sucrose diet (MASH diet) on the liver, male mice were fed with a MASH-inducing diet and received daily alcohol binges for three days, as shown in Figure 1A. The serum ALT and AST levels were significantly elevated in the MASH diet plus alcohol compared to the chow-fed mice (Figure 1B,C). However, we observed no change in the ALT levels in the mice fed on MASH diet or alcohol binges alone for three days, as shown in Supplementary Figure S1, suggesting it is the combined effect of a MASH diet and alcohol binges that causes increased liver injury. The hematoxylin–eosin (H&E) staining (Figure 1D) revealed increased steatosis in the mice with a MASH diet plus acute alcohol binges as compared to chow.

These data indicated that a short-term MASH diet in combination with acute alcohol binges promotes MetALD pathogenesis by increased liver damage.

### 3.2. Alcohol Binges and MASH Diet Leads to NLRP3 Inflammasome Activation

NLRP3 inflammasome activation is critical to the pathogenesis of chronic liver diseases such as ALD and MASH [13,16,17,18]. In addition to fibrosis and hepatic stellate activation in the MetALD murine model, NLRP3 inflammasome activation was shown to contribute to steatosis and inflammation in MASLD and ALD, respectively [25,35,36,37]. Therefore, next, we evaluated the activation of the NLRP3 inflammasome in the short-term feeding of a MASH diet plus alcohol binges model. NLRP3 inflammasome activation is a two-step process [10]. In the first priming step, there is an increase in the transcript level of NLRP3 and other inflammasome components, such as pro-IL-1b and pro-IL-18 [10]. In the second step, the protein level of cleaved caspase-1 and cleaved IL-1β is increased [10].

**Figure 2:**
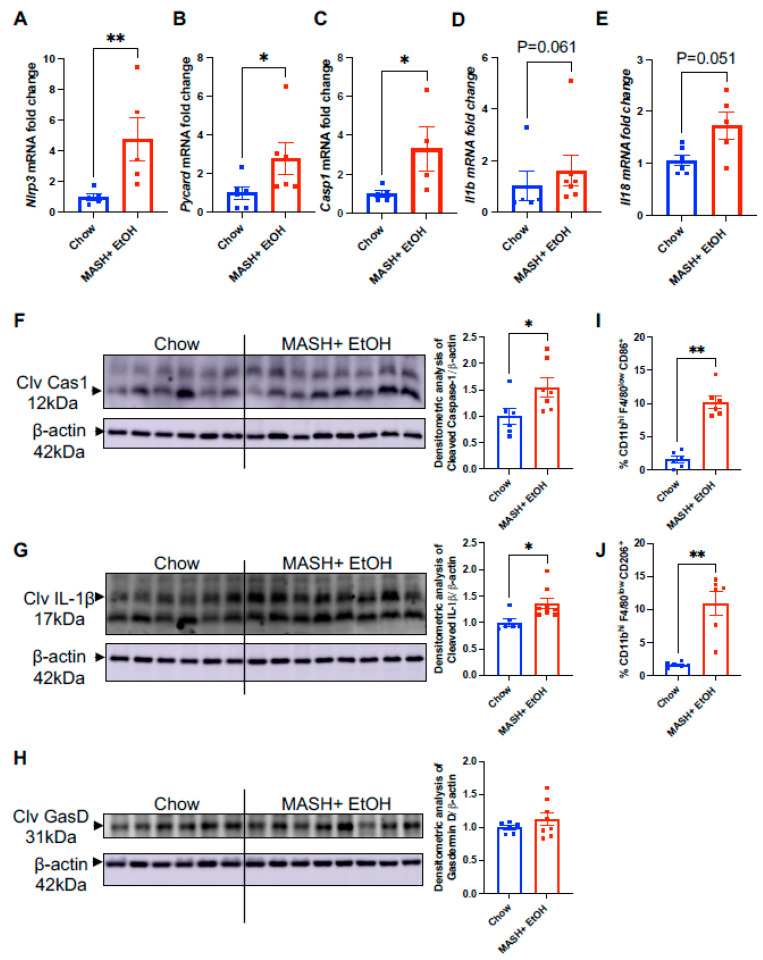
MASH diet and alcohol binges upregulate NLRP3 inflammasome. Liver RNA was used to determine *Nlrp3* (**A**), *Pycard* (**B**), *Casp1* (**C**), *Il1b* (**D**) and *Il18* (**E**) mRNA levels by qPCR. Here, 18s was used to normalize the Cq values. Liver lysates were used to detect cleaved caspase-1 (**F**), cleaved IL-1β (**G**) and cleaved gasdermin D (**H**) by Western blot. (**I**,**J**) Flow cytometry analysis of monocyte-derived macrophages, CD45^+^CD11b^hi^F4/80^low^CD86^+^, CD45^+^CD11b^hi^F4/80^low^CD206^+^. * *p* ≤ 0.05, ** *p* < 0.005.

We found a significant increase in the mRNA levels of *Nlrp3* (Figure 2A), apoptosis-associated Speck-like protein containing a caspase-1 recruitment domain (*Pycard*) (Figure 2B), pro-caspase-1 (*Casp1*) (Figure 2C), *Il1b* (Figure 2D), and *Il18* (Figure 2E) in the MASH diet plus acute alcohol binges compared to the chow diet. Furthermore, activation of the NLRP3 inflammasome was indicated by a significant increase in the cleaved caspase-1 and cleaved IL-Iβ in mice fed on the MASH diet plus acute alcohol binges compared to chow (Figure 2F,G). However, there was no significant difference in the gasdermin D levels in the chow and the MASH diet plus alcohol, suggesting that the NLRP3 inflammasome is not activated by a non-canonical pathway in the short-term feeding of the MASH diet plus acute alcohol binges (Figure 2H).

We also observed that the infiltrating monocyte-derived macrophages, CD45^+^CD11b^hi^F4/80^low^CD86^+^, CD45^+^CD11b^hi^F4/80^low^CD206^+^, were significantly increased in the liver in the MASH diet plus alcohol binge mice (Figure 2I,J). These results demonstrate that alcohol binges with the MASH diet promote NLRP3 inflammasome activation and inflammatory macrophages in the liver.

### 3.3. Short-Term Feeding of MASH Diet and Alcohol Binges Induce Hepatic Neutrophil Infiltration and NET Formation

**Figure 3:**
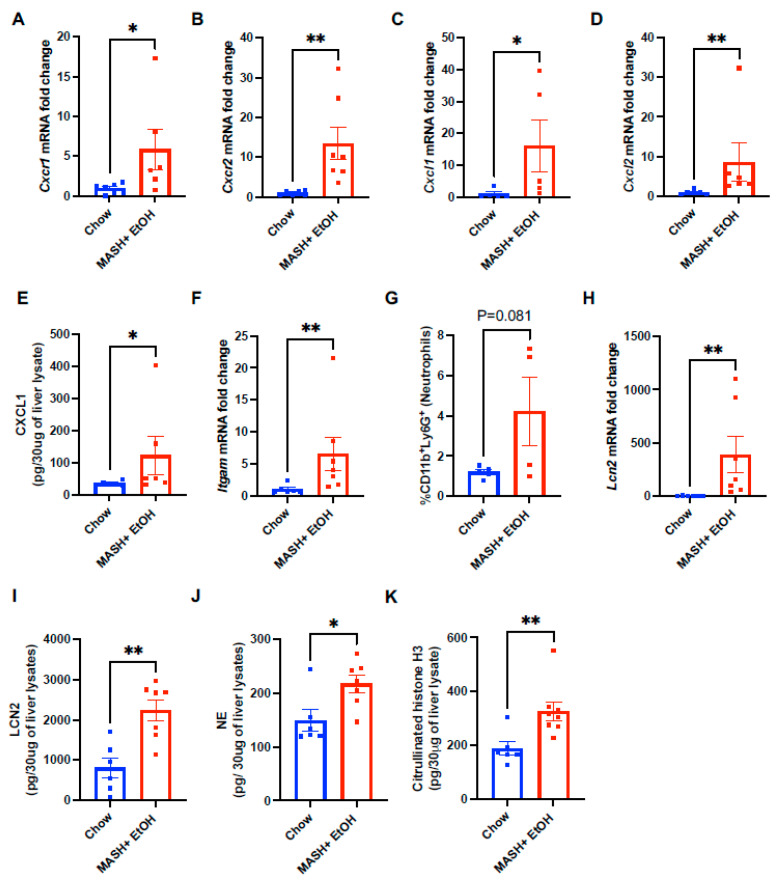
Short-term feeding of MASH diet and alcohol induces hepatic neutrophil infiltration and NET formation. Liver RNA was used to determine *Cxcr1* (**A**), *Cxcr2* (**B**), *Cxcl1* (**C**), *Cxcl2* (**D**), *Lcn2* (**H**) and *Itgam* (**F**) mRNA levels by qPCR. Here, 18s was used to normalize Cq values. Whole-cell liver lysates were used to detect CXCL1 (**E**) by ELISA. (**G**) Flow cytometry analysis of neutrophils (CD11b^+^Ly6G^+^) in liver immune cells. (**I**–**K**) Whole-cell liver lysates were used to detect LCN2 (**I**), NE (**J**), and Cit-H3 (**K**) by ELISA. * *p* ≤ 0.05, ** *p* < 0.005.

Alcohol binges have previously been shown to cause increased neutrophil infiltration, both in murine models of ALD and in patients [23,24,38]. Therefore, we next evaluated the level of chemokines involved in neutrophil infiltration. Hepatic expression of C-X-C Motif Chemokine Receptor 1 (*Cxcr1*) and C-X-C Motif Chemokine Receptor 2 (*Cxcr2*), which facilitates neutrophil infiltration via CXC chemokines, was significantly increased in the mice fed on a MASH diet plus acute alcohol binges as compared to chow (Figure 3A,B). Consistent with the increased chemokine receptor expression, we found a significant increase in the hepatic expression of neutrophil chemokines such as *Cxcl1* and *Cxcl2* (Figure 3C,D) in the MASH diet plus acute alcohol binges as compared to chow. Similarly, we found an increase in the level of CXCL1 in the liver lysates (Figure 3E).

In addition, the neutrophil marker *Itgam* (CD11b) was significantly increased in the mice fed on the MASH diet plus acute alcohol binges, which correlated with an increase in circulating neutrophils (CD45^+^CD11b^+^Ly6G^+^) in the MASH diet plus alcohol binge mice (Figure 3F,G).

Activated neutrophils, in response to microbial or endogenous signals, release NETs composed of decondensed chromatin, nuclear, and granular proteins [38,39]. We found a significant increase in the levels of the NET marker, LCN2, both in the liver mRNA and the lysates (Figure 3H,I). Neutrophil elastase (NE) (Figure 3J) and citrullinated histone 3 (CitH3) (Figure 3K) were also significantly increased in the livers of mice fed on the MASH-diet plus alcohol as compared to chow, indicating elevated NET formation in combined liver injury.

These results demonstrate that a MASH diet plus acute alcohol binges induce neutrophil recruitment, infiltration and NET release in the liver.

### 3.4. Inhibition of NLRP3 Inflammasome by MCC950 Ameliorates Liver Damage

**Figure 4:**
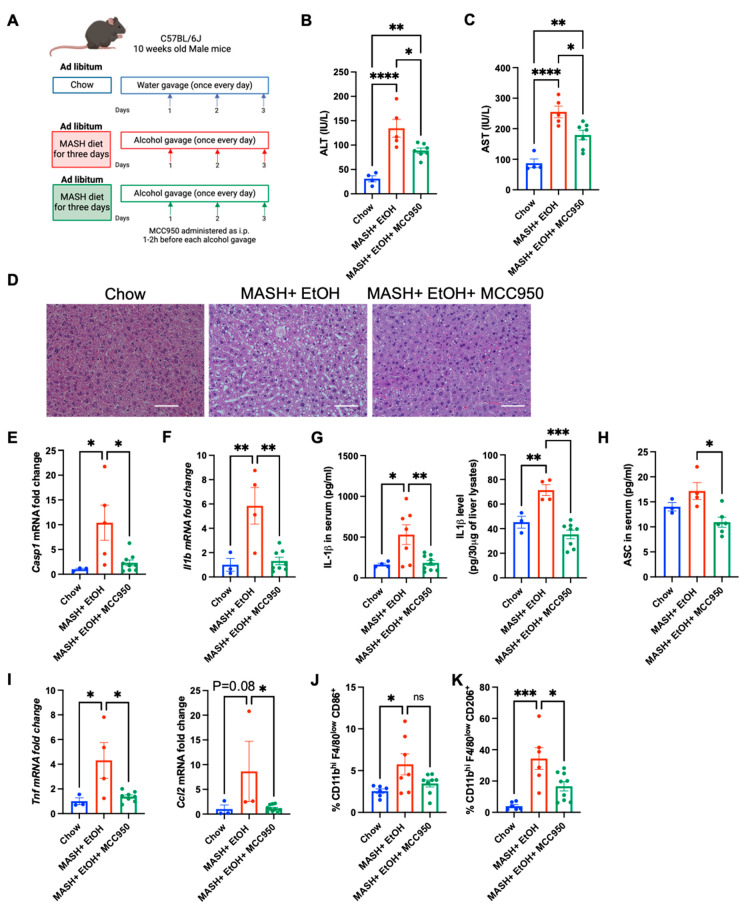
Inhibition of NLRP3 inflammasome by MCC950 ameliorates liver injury in short-term feeding of MASH diet and alcohol binges. (**A**) Feeding schematics for combined liver injury. C57BL/6 wild-type (WT) mice (n = 6–8) were fed with a MASH diet plus acute alcohol binges in the presence and absence of MCC950, with a standard laboratory chow diet as a control, for three days. (**B**,**C**) ALT and AST levels were measured from serum. (**D**) Formalin-fixed liver sections were stained with hematoxylin and eosin and representative slides are shown, scale bar = 50 μm. Liver RNA was used to determine *Casp1* (**E**), and *Il1b* (**F**), mRNA levels by qPCR. Here, 18s was used to normalize Cq values. (**G**) IL-1β level in the mice serum and whole-cell liver lysate was detected by ELISA. (**H**) ASC level in mice serum was detected by ELISA. (**I**) Liver RNA was used to determine *Tnf* and *Ccl2*, mRNA levels by qPCR. Moreover, 18s was used to normalize Cq values. Flow cytometry analysis of monocyte- derived macrophages, CD45^+^CD11b^hi^F4/80^low^CD86^+^ (**J**), CD45^+^CD11b^hi^F4/80^low^CD206^+^ (**K**). * *p* < 0.05, ** *p* < 0.005, *** *p* < 0.0005, **** *p* < 0.00005.ns; not significant.

To further corroborate the involvement of the NLRP3 inflammasome in the short-term feeding of a MASH diet plus acute alcohol binges, MCC950, an inhibitor of the NLRP3 inflammasome, was administered to the MASH diet-fed mice for three consecutive days, 1–2 h before the alcohol gavage (Figure 4A). The MASH diet plus acute alcohol binges elevated the ALT and AST levels significantly compared to the chow diet, and this was significantly attenuated in the mice that received MCC950 (Figure 4B,C). Hematoxylin–eosin (H&E) staining revealed decreased steatosis in the mice that received MCC950 with the MASH diet plus acute alcohol binges compared to the vehicle control (Figure 4D).

Inhibition of the NLRP3 inflammasome by MCC950 led to a significant reduction in the transcript levels of pro-caspase-1 (*Casp1*) (Figure 4E) and *Il1b* (Figure 4F). Furthermore, the levels of IL-1β in the serum and in the liver of mice fed on the MASH diet plus acute alcohol binges in the presence of MCC950 were significantly attenuated compared to the MASH diet plus acute alcohol binges alone (Figure 4G). Similarly, the ASC protein in the serum was significantly reduced in the presence of MCC950 compared to vehicle alone (Figure 4H), suggesting NLRP3 activation is dampened upon MCC950 administration in short-term feeding of a MASH diet plus acute alcohol binges.

Next, we analyzed whether the inhibition of the NLRP3 inflammasome reduces inflammation in short-term feeding of a MASH diet plus alcohol binges. The MASH diet plus acute alcohol binges induced an increase in the mRNA levels of *Tnf* and *Ccl2* (Figure 4I), which was significantly attenuated by MCC950 administration. Furthermore, the increase in infiltrating monocyte-derived macrophages, CD45^+^CD11b^hi^F4/80^low^CD86^+^ and CD45^+^CD11b^hi^F4/80^low^CD206^+^, was significantly reduced after MCC950 treatment in the mice fed on a MASH diet plus acute alcohol binges (Figure 4J,K). These results indicated that inhibition of NLRP3 inflammasomes ameliorates liver damage in short-term feeding of a MASH diet plus acute alcohol binges.

### 3.5. NLRP3 Inflammasome Inhibition Attenuates Neutrophil Infiltration and NET Formation

**Figure 5:**
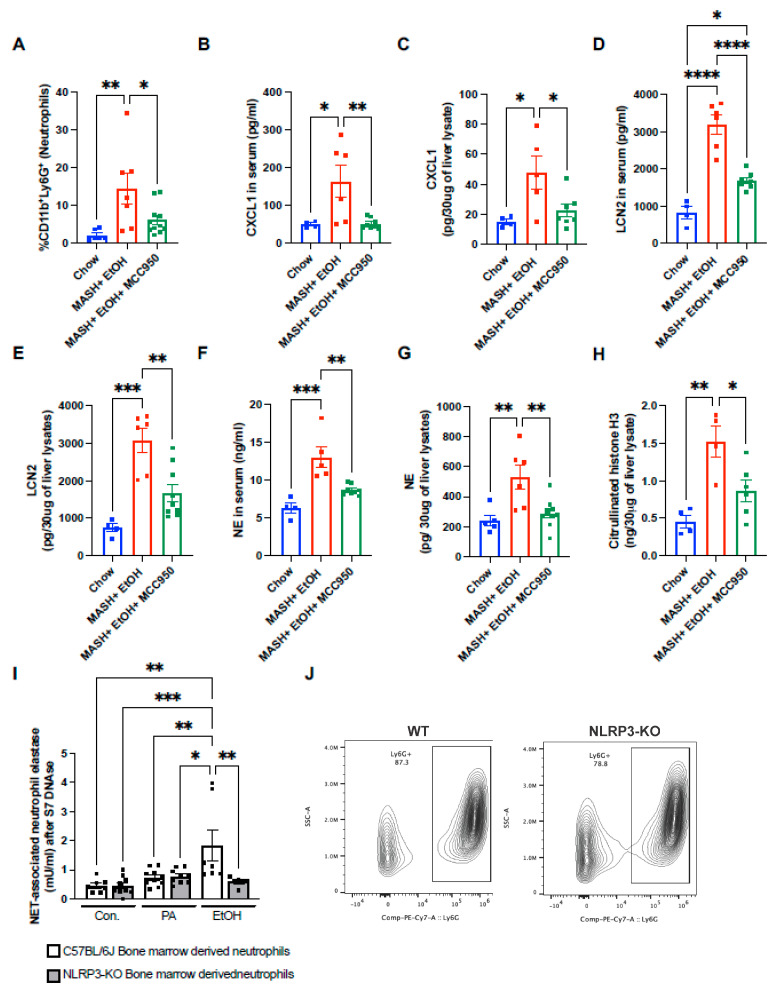
Inhibiting NLRP3 attenuates neutrophil infiltration and NET formation in short-term feeding of MASH diet plus alcohol binges. (**A**) Flow cytometry analysis of neutrophils (CD11b^+^Ly6G^+^) in liver immune cells. (**B**,**D**) CXCL1 and LCN2 were detected in serum by ELISA. (**C**,**E**) Liver lysates were used to detect CXCL1 (**B**), and LCN2 (**D**), by ELISA. (**F**) NE was detected in serum by ELISA. (**G**,**H**) Whole-cell liver lysates were used to detect the level of NE (**G**) and Cit-H3 (**H**) by ELISA. (**I**) Bone-marrow-derived neutrophils from WT and NLRP3-KO were treated with PA or EtOH, and NET-associated NE was measured to quantify NETs. (**J**) Contour plot depeciting the Ly6G^+^ cells. * *p* < 0.05, ** *p* < 0.005, *** *p* < 0.0005, **** *p* < 0.00005.

Given the observation that mice fed on a MASH diet plus acute alcohol binges exhibit increased neutrophil infiltration and NLRP3 inflammasome activation, we next questioned if the NLRP3 inflammasome is directly involved in promoting neutrophil infiltration and NET formation in the liver. Consistent with this observation, there was a significant reduction in CD45^+^CD11b^+^Ly6G^+^ infiltrating neutrophils in the mice administered MCC950 with a MASH diet plus acute alcohol binges (Figure 5A).

Next, we evaluated the level of CXCL1, which was previously shown to promote the recruitment of neutrophils to the liver. In the presence of MCC950, the CXCL1 protein level in both the serum and liver lysate was significantly reduced as compared to the MASH diet plus acute alcohol binges (Figure 5B,C). MCC950 administration resulted in a significant reduction in the LCN2 and NE protein, both in the serum and liver (Figure 5D–G). Similarly, another NET marker, CitH3, was also reduced in the MCC950-treated group compared to the vehicle (Figure 5H).

To further validate whether NET formation is dependent on NLRP3, the bone marrow neutrophils from WT and NLRP3-KO mice were isolated, and NETs were induced by either 50 mM alcohol or 330 μM palmitic acid (PA). Alcohol failed to induce NETs in the bone marrow neutrophils isolated from NLRP3-KO mice compared to the WT (Figure 5I). Over 80% of Ly6G^+^ neutrophils confirmed the purity of the neutrophils isolated from bone marrow cells (Figure 5J). These results highlight that the neutrophil expression of NLRP3 plays an indispensable role in NET formation.

### 3.6. Exogenous IL-1β Regulates Production of CXCL1 and LCN2 from Primary Hepatocytes

**Figure 6:**
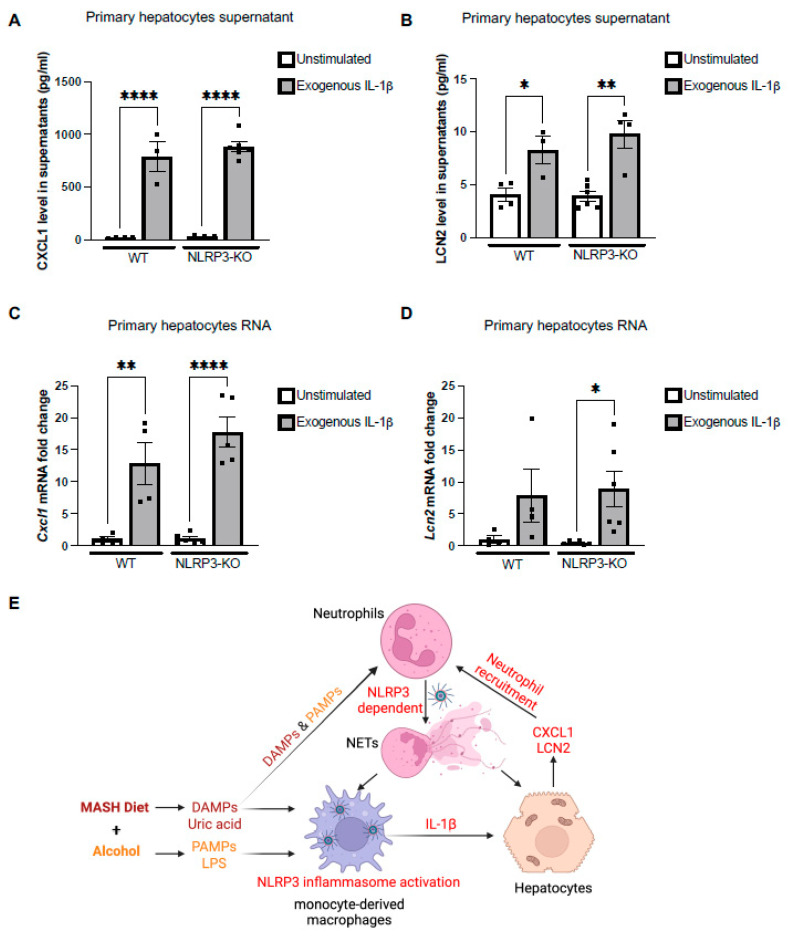
Exogenous IL-1β regulates production of CXCL1 and LCN2 from primary hepatocytes. (**A**,**B**) Supernatant of WT and NLRP3-KO primary hepatocytes after exogenous IL-1β treatment was used to detect CXCL1 (**A**) and LCN2 (**B**) by ELISA. mRNA of WT and NLRP3-KO primary hepatocytes after exogenous IL-1β treatment was used to determine *Cxcl1* (**C**), and *Lcn2* (**D**), mRNA levels by qPCR. Here, 18s was used to normalize Cq values. Whole-cell lysate of * *p* < 0.05, ** *p* < 0.005, **** *p* < 0.00005 (**E**) Briefly, MASH diet and alcohol binges lead to increased production of DAMPs and PAMPs, which activate NLRP3 inflammasome in monocyte-derived macrophages. NLRP3-dependent IL-1β promotes the production of CXCL1 and LCN2 from damaged hepatocytes, which in turn leads to increased neutrophil infiltration. NET formation by infiltrated neutrophils occurs in an NLRP3-depedent manner.

Because we found that NLRP3 inflammasome inhibition reduces the levels of CXCL1 and LCN2, we next asked if NLRP3 or its downstream signaling regulates the expression of CXCL1 and LCN2. The primary source of CXCL1 and LCN2 in the liver is hepatocytes. Using the primary hepatocytes isolated from WT and NLRP3-KO mice, we found an increase in CXCL1 and LCN2 production both at the protein (Figure 6A,B) and RNA levels (Figure 6C,D) by WT and NLRP3-KO primary hepatocytes after exogenous IL-1β treatment. Induction of CXCL1 and LCN2 in both WT and NLRP3-KO primary hepatocytes suggested that the production of these cytokines is dependent on the NLRP3 inflammasome-mediated increase in IL-1β; therefore, this highlighted the importance of the NLRP3-IL-1β axis in intercellular communication in the liver.

## 4. Discussion

NLRP3 activation has previously been shown to promote the disease progression in chronic ALD and in MASLD/MASH, respectively [13,16,17,18]. Here, we show that even a short-term combined insult of a MASH-inducing diet and alcohol binges activate the NLRP3-IL-1β axis in the liver. Our results also indicate the important role of intercellular communication between neutrophils and hepatocytes in this early phase of liver injury. The MASH diet plus alcohol binges resulted in infiltration of monocyte-derived macrophages as well as neutrophil recruitment to the liver, and this was associated with NLRP3 activation and IL-1β production. Our results suggest that both macrophages and neutrophils were activated via NLRP3 as MCC950, an NLRP3 inhibitor, prevented these effects. Furthermore, the lack of gasdermin D activation and the increase in only the NLRP3-IL-1β levels indicate the canonical NLRP3 activation. We show that IL-1β, a product of NLRP3 activation, acts on the hepatocytes by inducing CXCL1 and LCN2 that, in turn, further recruit and activate neutrophils in the liver. Our experiments with in vivo administration of the NLRP3 inhibitor, MCC950, show that the NLRP3 inhibition can block all of the early effects of a MASH diet and alcohol on the liver by preventing liver damage, steatosis, inflammation, neutrophil infiltration and NETs.

Previous studies by our group and others have shown that in both MASH and ALD, there is an increase in the circulating levels of DAMPs and PAMPs [14,31,40]. In ALD, the increase in uric acid and ATP from damaged hepatocytes activates the NLRP3 inflammasome, which facilitates the induction of IL-1β and TNF-α by immune cells [40]. Similarly, in MASH, uric acid and high-mobility group box 1 protein (HMGB1) are increased in the early phase, and with subsequent progression of the disease, the level of endotoxin and ATP increases [31]. In ALD and MASLD/MASH, intestinal dysbiosis/gut leakiness is a common phenomenon. Therefore, increased serum LPS is found in mouse models as well as in human subjects of both diseases [40,41,42]. Thus, in our model, we propose that a combination of DAMPs and PAMPs, induced by the combined MASH diet and alcohol, activates the NLRP3 inflammasome in monocyte-derived macrophages and neutrophils.

MASH diet results in steatosis in 8 weeks and mice develop early fibrosis at 27 weeks [31]. At 8 weeks, there is only a slight induction of the NLRP3 inflammasome and its components at the mRNA level [31]. Interestingly, it was only male mice that developed robust steatohepatitis and showed activation of the inflammasome at 16 weeks. However, in the short-term feeding of a MASH diet and acute alcohol binges, we observed an increase in the NLRP3 inflammasome components at both the RNA and protein levels after three days in male mice [30]. This further implies that in the early phase of MetALD, similar triggers as described in chronic models of MASH and ALD induce canonical NLRP3 activation and accelerate liver damage. Upon NLRP3 activation, the release of ASC specks drives the pathogenesis in MASH by activating HSCs [43], and in ALD, circulating ASC specks perpetuate inflammation and contribute to the progression of the disease [19]. In our early MetALD model, an increase in the circulating ASC, which was attenuated after MCC950 administration, may perpetuate the increased inflammation observed in this model.

A previous study on the short-term feeding of HFD and single alcohol binge highlighted the increased expression of CXCL1, which led to increased neutrophil infiltration in the liver [26]. Another study in hepatic ischemia liver injury showed a potential correlation between neutrophil infiltration and NLRP3 [44]. Our findings suggest that NLRP3 is directly involved in neutrophil infiltration in the liver. We discovered that alcohol-induced NET formation was dependent on NLRP3 expression in neutrophils, as bone-marrow-derived neutrophils isolated from NLRP3-KO mice did not exhibit alcohol-induced NETosis. We also demonstrated that NLRP3 inflammasome inhibition attenuated the neutrophil infiltration and NET formation in the early phase of MetALD.

In type 2 diabetes mellitus, IL-1β has previously been shown to regulate the recruitment of NF-κB and STAT1 transcription factors to the promoter region [45]. In this model, we observed that IL-1β, induced by the NLRP3 activation, has a perpetuating effect on the liver inflammation. We found that, in hepatocytes, IL-1β induces key chemokines, such as CXCL1 and LCN2, involved in neutrophils recruitment. This effect of IL-1β was independent of the NLRP3 expression in hepatocytes, highlighting the role of immune cell-derived NLRP3 activation and IL-1β in intercellular crosstalk in the liver.

In conclusion, our study highlights the novel role of the NLRP3-IL-1β axis in the early phase of MetALD in propagating inflammation by recruiting monocytes/macrophages and neutrophils to the liver. Our study demonstrates that the intercellular communication between monocytes, hepatocytes and neutrophils is orchestrated by the NLRP3-IL-1β axis and therapeutic inhibition of NLRP3 in the early phase of MetALD prevents liver damage, inflammation and steatosis (Figure 6E).

## Figures and Tables

**Table 1:** Sequences of real-time PCR primers (Mouse SYBR Green primers).

Gene	Forward Sequence (5′-3′)	Reverse Sequence (5′-3′)
*Casp1*	GGCACATTTCCAGGACTGACTG	GCAAGACGTGTACGAGTGGTTG
*Ccl2*	TCTTGGTTCCCTGGCGTACTCT	GTGAGTGTCACTCTCCAGTTTGC
*Cxcl1*	CAAGGCTGGTCCATGCTCC	TGCTATCACTTCCTTTCTGTTGC
*Cxcl2*	CCAACCACCAGGCTACAGG	GCGTCACACTCAAGCTCTG
*Cxcr1*	TCTGGACTAATCCTGAGGGTG	GCCTGTTGGTTATTGGAACTCTC
*Cxcr2*	TGTCTGGGCTGCATCTAAAGT	AGGTAACCTCCTTCACGTATGAG
*Il18*	CCTACTTCAGCATCCTCTACTGG	AGGGTTTCTTGAGAAGGGGAC
*Il1b*	TGGACCTTCCAGGATGAGGACA	GTTCATCTCGGAGCCTGTAGTG
*Itgam*	ATGGACGCTGATGGCAATACC	TCCCCATTCACGTCTCCCA
*Lcn2*	TGGCCCTGAGTGTCATGTG	CTCTTGTAGCTCATAGATGGTGC
*Nlrp3*	TCACAACTCGCCCAAGGAGGAA	AAGAGACCACGGCAGAAGCTAG
*Pycard*	CGGAAAGTGGAATCCTTGCAGG	AGCAGTGAGGTCAGGCTTGGAA
*Tnf*	CTGGATGTCAATCAACAATGGGA	ACTAGGGTGTGAGTGTTTTCTGT

## Data Availability

All the data relevant to the study are included in the article or uploaded as supplementary information.

## Supplementary Materials

The following supporting information can be downloaded at: https://www.mdpi.com/article/10.3390/cells13110960/s1. Figure S1. Short-term feeding of alcohol binges, MASH diet and MASH diet plus acute alcohol binges.

## Abbreviations

AH	Alcoholic hepatitis/Alcohol-associated hepatitis
ALD	Alcoholic liver disease/Alcohol-associated liver disease
ALT	Alanine aminotransferase
ASC	Apoptosis-associated speck-like protein containing a caspase activating and recruitment domain
AST	Aspartate aminotransferase
CCL2/MCP-1	chemokine (C-C motif) ligand 2/Monocyte chemoattractant protein 1
Cit-H3	Citrullinated histone 3
CXCL1	Chemokine (C-X-C motif) ligand 1
CXCL2	Chemokine (C-X-C motif) ligand 2
CXCR1	C-X-C Motif Chemokine Receptor 1
CXCR2	C-X-C Motif Chemokine Receptor 2
DAMPs	Damage-associated molecular patterns
HF-HC-HS	High fat–cholesterol–sucrose diet
HFD	High-fat diet
HSC	Hepatic stellate cell
IL-1β	Interleukin 1 beta
IL-18	Interleukin 18
LCN2	Lipocalin 2
LMNCs	Liver mononuclear cells
MASH	Metabolic-dysfunction-associated steatohepatitis
MASLD:	Metabolic-dysfunction-associated steatotic liver disease
MFI	Mean fluorescent intensity
NE	Neutrophil elastase
NET(s)	Neutrophil extracellular trap(s)
NLRP3	Nod-like receptor protein 3
PAMPs	Pathogen-associated molecular pattern molecules
TLR	Toll-like receptor
TNF-α	Tumor necrosis factor alpha
