# Supplementary material for: Combined Insults of a MASH Diet and Alcohol Binges Activate Intercellular Communication and Neutrophil Recruitment via the NLRP3-IL-1β Axis in the Liver
Source: Cells. 2024 Jun 1;13(11):960. doi: 10.3390/cells13110960 (PMC11171595; doi:10.3390/cells13110960)
Supplement: Supplementary file 1 [file cells-13-00960-s001.zip › cells-2994254-supplementary.pdf]

Combined insults of MASH diet and alcohol binges activate intercellular communication and neutrophil recruitment via the NLRP3-IL-1β axis in the liver

Mrigya Babuta[1], Prashanth Thevkar Nagesh[1], Aditi Ashish Datta[1], Victoria Remotti[1], Yuan Zhuang[1], Jeeval Mehta[1], Francesca Lami[1], Yanbo Wang[1], Gyongyi Szabo[1,*]

[1]Department of Medicine, Division of Gastroenterology, Beth Israel Deaconess Medical Center and Harvard Medical School, Boston, MA 02215, USA

***Corresponding author:**

Name: Gyongyi Szabo, M.D., Ph.D.

Address: 330 Brookline Avenue, ST-214B, Boston, MA 02215, USA.

Phone: (617) 667-9050

Email address: gszabo1@bidmc.harvard.edu

KEY WORDS:

NETs, MetALD, monocyte/macrophage, steatosis, inflammation

SHORT TITLE: NLRP3-IL-1β axis in intercellular communication in MetALD

Supplementary data

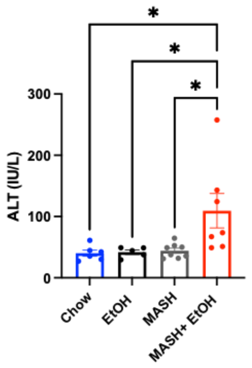

Figure S1: Short-term feeding of alcohol binges, MASH diet and MASH diet plus acute alcohol binges (A) ALT levels were measured from serum of chow, alcohol alone, MASH diet alone and MASH plus alcohol fed mice. * p<0.05
